# A Bibliometric Analysis of the Health Field Regarding Social Networks and Young People

**DOI:** 10.3390/ijerph16204024

**Published:** 2019-10-21

**Authors:** Pilar Aparicio-Martinez, Alberto-Jesus Perea-Moreno, María Pilar Martinez-Jimenez, María Dolores Redel-Macías, Manuel Vaquero-Abellan, Claudia Pagliari

**Affiliations:** 1 Grupo Investigación epidemiológica en Atención primaria (GC-12) del Instituto Maimónides de Departamento de Enfermería, Campus de Menéndez Pidal, Universidad de Córdoba, 14071 Córdoba, Spain; mvaquero@uco.es; 2 Usher Institute of Population Health Sciences and Informatics, University of Edinburgh, Edinburgh EH8 9YL, UK; 3 Grupo Investigación epidemiológica en Atención primaria (GC-12) del Instituto Maimónides de Investigación Biomédica de Córdoba (IMIBIC), Hospital Universitario Reina Sofía, 14071 Córdoba, Spain; 4 Departamento de Física Aplicada, Campus de Rabanales (ceiA3), Universidad de Córdoba, 14071 Córdoba, Spain; g12pemoa@uco.es (A.-J.P.-M.); fa1majip@uco.es (M.P.M.-J.); 5 Departamento Ingeniería Rural, Ed Leonardo da Vinci, Campus de Rabanales, Universidad de Córdoba, 14071 Córdoba, Spain; ig1remam@uco.es; 6 eHealth Research Group, Usher Institute of Population Health Sciences and Informatics, University of Edinburgh, Edinburgh EH8 9YL, UK; Claudia.Pagliari@ed.ac.uk

**Keywords:** social networks, health, young people, bibliometric study

## Abstract

Social networks have historically been used to share information and support regarding health-related topics, and this usage has increased with the rise of online social media. Young people are high users of social media, both as passive listeners and as active contributors. This study aimed to map the trends in publications focused on social networks, health, and young people over the last 40 years. Scopus and the program VOSviewer were used to map the frequency of the publications, keywords, and clusters of researchers active in the field internationally. A structured keyword search using the Scopus database yielded 11,966 publications. The results reveal a long history of research on social networks, health, and young people. Research articles were the most common type of publication (68%), most of which described quantitative studies (82%). The main discipline represented in this literature was medicine, with 6062 documents. North American researchers dominate the field, both as authors and partners in international research collaborations. The present article adds to the literature by elucidating the growing importance of social networks in health research as a topic of study. This may help to inform future investments in public health research and surveillance using these novel data sources.

## 1. Introduction

The creation of social groups to exchange information, share experiences, or provide support is a natural human impulse [1]. The growth of the internet has led to new channels for social networking, which have evolved and adapted to meet the needs and resources of the population [2].

In the digital era, online social networks have become a central node through which individuals connect and interact with other people [3], by sharing, viewing, or commenting on ideas and content posted by other users [4,5]. The use of social media has exponentially escalated since the late 1990s. The dynamic nature of these platforms has been the reason for their rapid growth, and the structure of these media has facilitated the creation of relationships among users [6,7]. Although individuals often use these networks to meet new people, there is a tendency to connect with those who hold similar expectations or preferences [8].

Additionally, one of the reasons for creating these social networks and exchanging information is to understand health, either from an individual or communal perspective [9]. Within these networks, young people are the most digitally connected members, both as active and passive users [10]. Nevertheless, adolescents and early adults are in a critical life stage, in which both self-identity and healthy or unhealthy behaviors are shaped [8,11]. Mental health issues such as depression, and physical disorders such as sexual infections, are more common in this group [11,12,13,14,15].

Recent research on this topic has focused on the relationship between social networks and health issues, both as prevention or educational tools, and as risk factors [6,16]. In this sense, researchers have explored the health-damaging effects of social media [5,15,17], or its side effects, such as isolation, depression, and eating disorders [18,19]. Different factors, such as gender or cultural background, have been linked to these side effects [10,12,20].

Other studies have explored the beneficial use of these networks for delivering health interventions [14,17], especially health education [21,22]. Engaging patients in health communities is also a topic of research, often focused on specific health problems or social support [22].

**Figure 1:**
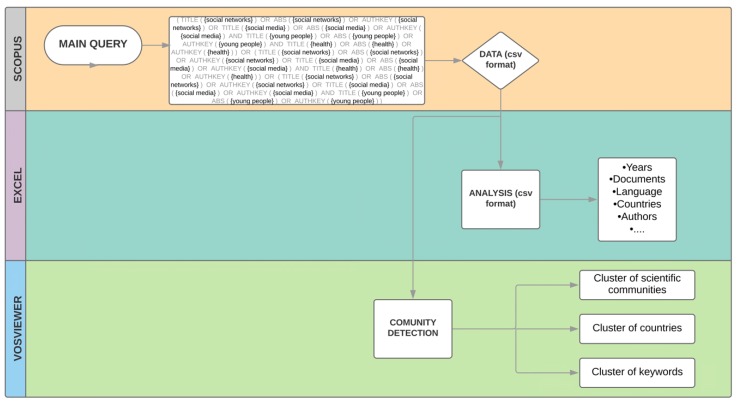
Methodology structure.

Overall, social media appears to have been used in different ways, depending on the user’s health and behavior [23,24]. Based on this, a previous study was carried out in Scopus using the terms “social media”, “health”, and “young people”. From this initial research (1785 documents), more recent publications and those published in journals with a high impact factor were used to represent the increase in the reach of social media and health (Table 1). Table 1 summarizes some of the latest publications regarding social networks as health problems or interventions. This research focused on the latest publications in major journals in the health field, such as the *Journal of Medical Internet Research* [25]. In this sense, the results showed that the health and education area tend to focus on the positive outcomes of using social networks. Meanwhile, the psychology area tends to study the side effects of using social media (Figure 1).

With this background, the principal objective of the present paper was to determine the tendencies of publications focused on social networks applied to health during the last 40 years (from 1978 to 2018). Additionally, the second objective of this study was to determine the link between social networks, health, and young people. The purpose of these objectives was to better understand the interaction of social networks in health, in order to assist the decision-making of health professionals and contribute to effective health education.

## 2. Research Approach

The analysis of previous works is an essential step in research in any field, though it is of great importance in the health field. This importance relates to the fact that new results contribute to the healthcare of patients. Additionally, this type of analysis has become a complementary tool to determine the quality of new scientific knowledge, and its impact on the health of the population. In this sense, it is possible to access the scientific data, and their effect on studies and sources [36].

Bibliometric studies provide essential information regarding the scientific data within a country, as well as in the international context. All of this information facilitates the decision-making of health professionals and will impact the future of social networking regarding health.

### 2.1. Database Selection

Prior to the analysis of the data from the research strategy, using the terms “social networks”, “health”, and “young people”, a comparative analysis between different databases was conducted. The research strategy used was ALL = (“social networks” AND “health” AND “young people”). The databases included in this analysis were Scopus, Web of Sciences (WOS), PubMed, the Health and Medical Collection, and the Psychology Database. These databases were included based on their importance, use, and relevance in the health field, and were used to compare the results with the initial research.

The exclusion criteria used were the period of time from 1987 to 2018, terms in all cases and document types, and excluding papers with no scientific relevance such as news, obituaries, projects, or patents, available in journals.

The results show that for WOS, the number of documents was similar to the results obtained using Scopus. The results of the research using PubMed showed fewer publications than the number of documents. The results from the research using the Health and Medical Collection and Psychology Database show a higher number of documents than Scopus. The significant difference between these databases compared to Scopus or WOS may be caused by the nature of these resources. The Health and Medical Collection and Psychology Database were created to include all content in any form, so as to improve the learning, teaching, and research needs of institutions. Thus, these databases include both scientific and less scientific documents, such as medical reference eBooks, instructional videos, dissertations, and working papers. These platforms also include thousands of evidence-based articles and clinical trial records [37].

Overall, the documents obtained using Scopus included most of the scientific productions in the topic of health, social networks, and young people. This is based on the fact that, when it was used for the same strategy research, which focused on all fields, Scopus included more results than the other databases.

### 2.2. Data Collection

For this study, Elsevier’s Scopus database was used to carry out the analysis. We identified studies from 1978 to 2018 that referred to social networks, health, or young people.

Scopus is a scientific bibliographic database of items from scientific journals. This database has been claimed as “the largest index database”, including up to 65 million records and claims, many of which are in the health field, with titles providing complete coverage of Medline, Embase, and Compendex. In addition to articles, this database includes series, conferences, papers, books, and patents. The sources in the database date back to 1823, and it was established in 1996. Moreover, Scopus also provides the performance status of papers and authors according to the citations received for each work [38,39].

For this research, the inclusion criteria were the period from 1978 to 2018, and the theme of social media and health.

### 2.3. Statistical Analysis

The results from the research were analyzed, focusing on descriptive analyses, such as the frequencies of the types of document, the language, trends in scientific publications, primary sources, the field of the publication, the leading scientific institutions, associations among nations, the primary authors in the area, and the keywords used. In the case of keywords, a normalization of the terms was carried out, as many of the main keywords had both singular and plural forms. The keywords included in the manuscript were the author’s keywords. In this sense, the keywords used were not MeSH terms.

Another aspect of the analysis was the identification of networks using the VOSviewer software [40]. This open-source program was created for constructing and viewing bibliometric maps by importing the data from several sources, including Scopus [41]. The criteria used to create the maps were a minimum of 10 connections between authors, fewer than 10 authors per document, and a minimum of five authors per document. This strategy was followed for the concurrency of keywords, connections between authors, and countries.

### 2.4. Exclusion and Inclusion Criteria

The inclusion criteria used for this study focused on the words “social networks” or “social media”, “health”, and “young people”. These terms were used based on the objectives of this study, as the purpose was to analyze the intervention of social networks in health. With the results from the terms “social networks” or “social media” and “health”, researchers looked for positive and negative interactions or applications of these networks to health. Additionally, the term “young people” was used to identify this specific population, determine implications, and find previous studies focused on this group.

Other terms, such as “youth” or “young adults”, were not included, as it would result in the inclusion of more data that were not adequately focused on young people. The boolean operators used were “OR” and “AND”, to link the three terms.

The exclusion criteria used were the period of time for the production of the documents, and the use of terms focusing on the title, abstract, or keywords. Additionally, the type of document was determined in order to exclude non-scientific productions, such as obituaries.

### 2.5. Sectional Analysis of the Initial Research Strategy

Before the use of the research strategy and the analysis of the data, the research strategy was divided into three sections. Each of these sections focused on the different relationships between health, social media, and young people.

The first section focused on the relationship between social media and the health of young people. The search used was (TITLE ({social networks}) OR ABS ({social networks}) OR AUTHKEY ({social networks}) OR TITLE ({social media}) OR ABS ({social media}) OR AUTHKEY ({social media}) AND TITLE ({young people}) OR ABS ({young people}) OR AUTHKEY ({young people}) AND TITLE ({health}) OR ABS ({health}) OR AUTHKEY ({health})) OR (TITLE ({social networks})). This strategy resulted in 262 documents, with the earliest publication in 1999. The most common theme in terms of the number of publications was medicine (166), followed by social sciences (83).

The second section was based on the interaction between social media and health. The search used was the following: (TITLE ({social networks}) OR ABS ({social networks}) OR AUTHKEY ({social networks}) OR TITLE ({social media}) OR ABS ({social media}) OR AUTHKEY ({social media}) AND TITLE ({health}) OR ABS ({health}) OR AUTHKEY ({health})). This second search resulted in 10,900 documents, with 5917 from the medicine area and 2750 from the social sciences thematic area.

The third section focused on the connection between social networks and young people. The search used was (TITLE ({social networks}) OR ABS ({social networks}) OR AUTHKEY ({social networks}) OR TITLE ({social media}) OR ABS ({social media}) OR AUTHKEY ({social media}) AND TITLE ({young people}) OR ABS ({young people}) OR AUTHKEY ({young people})). From this research, 1320 documents were found, with the first dated in 1997. The area with the most publications was social sciences (794), followed by medicine (305). Additionally, the results from this search were further analyzed using NOT (TITLE (“health”) OR ABS ({health}) OR AUTHKEY ({health})). This deeper analysis showed that 25 documents did not include the term health, though the thematic areas were first medicine (17 documents), and then social sciences (11 documents).

Based on the results from each section, the final strategy was as follows: (TITLE ({social networks}) OR ABS ({social networks}) OR AUTHKEY ({social networks}) OR TITLE ({social media}) OR ABS ({social media}) OR AUTHKEY ({social media}) AND TITLE ({young people}) OR ABS ({young people}) OR AUTHKEY ({young people}) AND TITLE ({health}) OR ABS ({health}) OR AUTHKEY ({health})) OR (TITLE ({social networks}) OR ABS ({social networks}) OR AUTHKEY ({social networks}) OR TITLE ({social media}) OR ABS ({social media}) OR AUTHKEY ({social media}) AND TITLE ({health}) OR ABS ({health}) OR AUTHKEY ({health})) OR (TITLE ({social networks}) OR ABS ({social networks}) OR AUTHKEY ({social networks}) OR TITLE ({social media}) OR ABS ({social media}) OR AUTHKEY ({social media}) AND TITLE ({young people}) OR ABS ({young people}) OR AUTHKEY ({young people})). This, based on the health field, connected the terms “social networks”, “health”, and “young people”.

The data obtained was a .csv file that contained the following: authors, title, author IDs, year, volume, issue, source title, article number, number of pages, cited by, digital object identifier system (DOI), link, document type, access type, source, and ID. Each item from the previous step was analyzed and studied separately; for instance, the number of documents per country, or the rate of publication of each author. Finally, the cluster determination of the thematic collections was examined with VOSviewer, resulting in diverse maps of global connections between authors and countries, as well as research tendencies, using keywords (Figure 1).

## 3. Results and Discussion

Article frequency, disciplinary focus, topics, authors’ institutional affiliation, and country are all useful indicators of the popularity and type of research being undertaken in a scientific field, as well as its trends.

### 3.1. Type and Language of the Works

**Figure 2:**
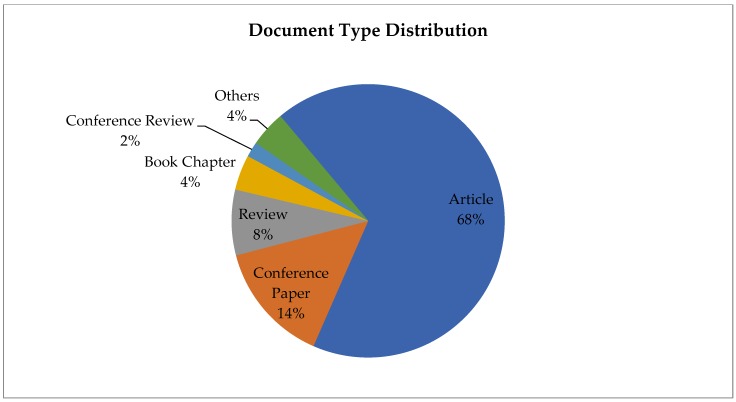
Frequency of the types of documents from 1978 to 2018.

At total of 11,966 documents were obtained for the period of 1978–2018. Publications were diverse in type; the most common type of document was articles (68%), followed by conference papers (14%). The remaining types were reviews (8%); book chapters (4%); conference reviews (2%); and other types of documents (4%), such as books or notes (Figure 2). For the most common document, articles, the frequency was studied, and it was found that 82% were quantitative studies and 18% were qualitative studies. Most of the quantitative studies were cross-sectional studies (24%), followed by control trial studies (23%). These results are consistent with previous studies that have pointed out how quantitative articles are more common in the health field, with reviews or other documents being less commonly published [42]. As described by van Wesel M. (2016), the reasons for the higher number of articles may be related to a change of publication policy, author interest, or hot topic issues [43].

**Figure 3:**
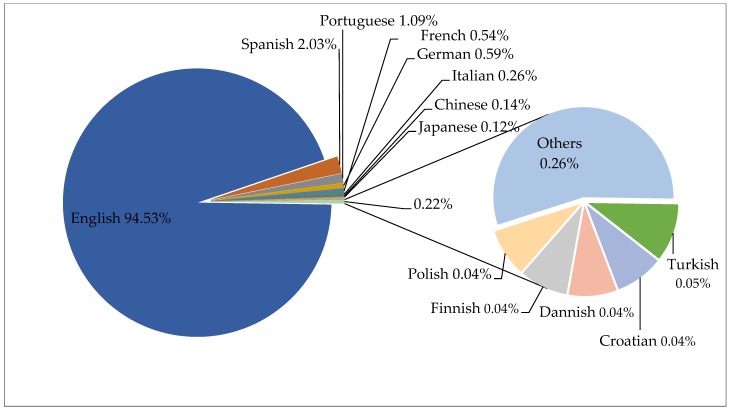
Languages of papers published over the period of 1978 to 2018.

Regarding the language used in the publications found in the search, the language most used was English in the different international journals (94.53%), followed by Spanish (2.03%), Portuguese (1.09%), and German (0.59%). Figure 3 shows the frequency of each language for the documents published over the last four decades, as found through the bibliometric examination.

The tendency to use English has been described in previous studies as the main language of publication [44], noting that researchers who write in English to communicate tend to have more opportunities [45].

### 3.2. Characteristics of Scientific Productions from 1978 to 2018

**Figure 4:**
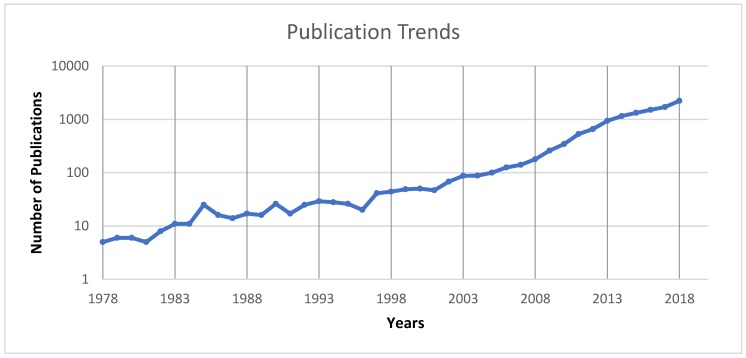
The trend of publications in social networks, health, and young people during the period of 1978–2018.

Figure 4 shows the frequency of academic publications focused on social networks, health, and young people, over the last four decades. The figure suggests an upward trend, implying that the number of annual outputs increased markedly from around 2002 to 2018.

Based on this figure, the main observation is a rapid increase from the early 2000s, which coincides with the emergence of online social media and research exploring the interaction between social media and health.

These results are consistent with previous analyses showing increased research attention given to social networks related to the health field [34]. This interest highlighted the possibility of using these networks as tools, but also their negative effects on health [46]. Additionally, it is important to highlight that the increase of publications also affects other topics in the health field. In this sense, Kyvik S (2003) highlighted how the number of publications per researcher was higher in technology and the natural and medical sciences in 1998–2000. Additionally, this same author stated that the tendency for publication in such areas increased in the late 1990s [47].

### 3.3. International Dissemination of Publications

**Figure 5:**
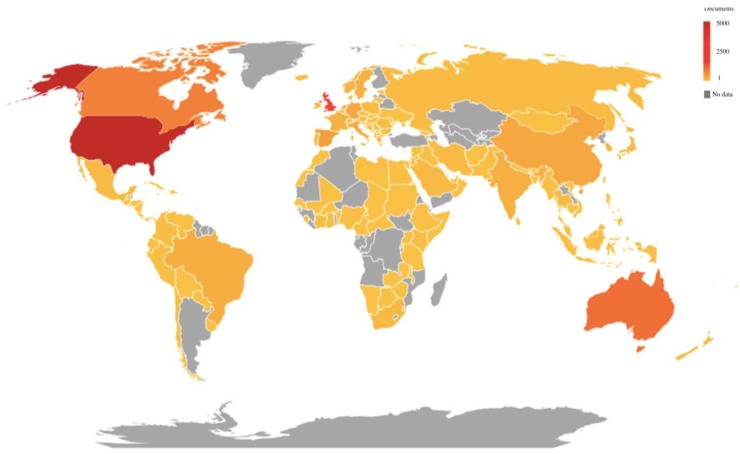
The trend of publications in each country during the period of 1978–2018.

Figure 5 shows the production of relevant articles per country between 1978 and 2018. Colors indicate the number of papers, from red (highest) to grey (no publications). The country with most publications was the United States (5205), followed by the United Kingdom (1577), Australia (1058), Canada (811), and Spain (423). Within these countries, the use of social networks has increased, and has even been potentiated by governments and institutions in order to promote healthy lifestyles or to provide group support for patients [48]. In the case of Spain, the increase of publications related to social media and health might be linked to the growth in environmental performance, social performance, and corporate governance performance since 2002 [49].

**Figure 6:**
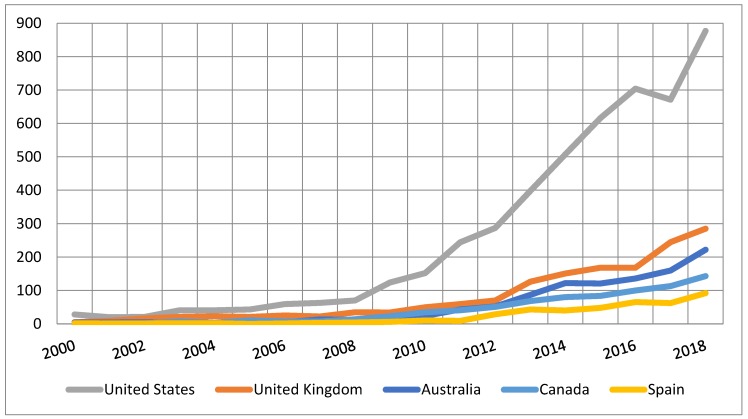
The trend of publications in the five countries with higher rates during the period of 1978–2018.

Figure 6 shows the trajectory of research publications in each of the five countries with the highest production of papers on social networks, young people, and health, revealing the highest increase occurred in the United States.

**Figure 7:**
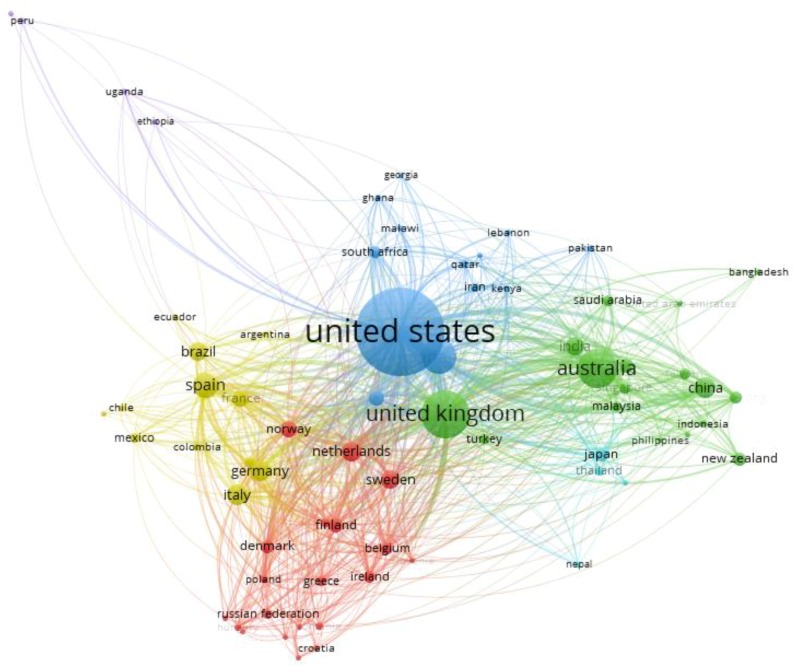
Collaboration among countries.

The social network map shown in Figure 7 illustrates the pattern of international collaboration between study authors. This figure was obtained after applying the software VOSviewer v.1.6.11. to a .csv file of the data extracted from Scopus during the literature search.

Three countries dominate in the six clusters seen in Figure 7 and Table 2, namely: the United Kingdom, the United States, and Australia. The first cluster comprises eastern European countries and Nordic countries, led by Finland. The green cluster, which is the second most crucial cluster, is led by the United Kingdom. The United Kingdom is the node of this cluster, because of the number of connections with other countries, and the number of publications. This cluster also includes Australia as the second most relevant nucleus, with a lower number of connections than the United Kingdom. All of the countries from this cluster seem to be connected via economic and political relationships.

The blue cluster is third in importance, and is led by the United States, followed by Canada, and represents 24.2% of the publications. The yellow cluster is led by Spain, with connections to Latin America and Europe, representing 13.4% of publications on this topic. The purple cluster is linked to Latin America and African countries, led by Cuba. The last cluster is pink and is led by Japan, and is connected to a variety of different countries such as the United Kingdom, the United States, Australia, and New Zealand.

Of the countries that have published the most about social media in the health field, the United States stands out. Previous researchers have stated that the United States has dominated publications in different scientific fields, such as education. This tendency of publications to originate from the United States and a few other countries, such as the United Kingdom, has been attributed to a combination of factors, such as being English-speaking countries, authors coming from these countries, and the possible connections between researchers within the scientific community [50]. These results and previous works further support the idea of the United States being the leader of scientific productions in the health field, and therefore in the topic of social media connected to health and young people.

The essential and significant role of the United States is also shown by the connections between authors and affiliations, most of which belong to the United States (Table 3). Overall, these results might be explained by the fact that there may be economic, historical, geographical, and cultural influences between the groups, which can be applied to all of the clusters. In addition, the remaining clusters could be explained by specific topics relating to social networks, such as interventions or risks, and the type of young people that the research focused on.

### 3.4. Institutions Active in Relevant Research

In Table 3, the 10 organizations with the highest rates of publication in the field of social networks related to health and young people are presented. Additionally, the top three keywords used in each of these institutions are included in this table.

The University of Toronto is in first position, with 158 documents, which is not surprising, as the *Journal of Medical Internet Research* is based at this location. Next is the University of Sydney, in second position with 157 documents, and the University of Michigan in third position with 155. In positions four–six are the University of North Carolina at Chapel Hill with 152, the University of Washington with 143, and the University of Melbourne with 140 documents published. Finally, Harvard Medical School has 132, University of California has 131, Johns Hopkins Bloomberg School of Public Health has 126, and the University of California has 123. It should be highlighted that the keyword used most often by these institutions is “human/s”, ranking in first place in all cases.

The increase of publications and the ranking of affiliations might be related to collaboration between authors. These collaborations have been previously studied by other authors, showing that, since 1997, collaborations in the United States or Canada have increased by 20% [51]. Moreover, these factors have been linked to collaborations between the United States and the other countries, showing a possible node of union [51].

Regarding the type of study implemented by each institution, according to Scopus, the results showed that all of the institutions focused on articles in the area of medicine, followed by the area of social sciences. The central countries with a higher number of publications were the United States, United Kingdom, and Canada. Finally, the most common keywords used according to Scopus were “human/s”, “female”, “articles”, “male”, and “social media.”

### 3.5. Subject Categories and Journals Found using Scopus

**Figure 8:**
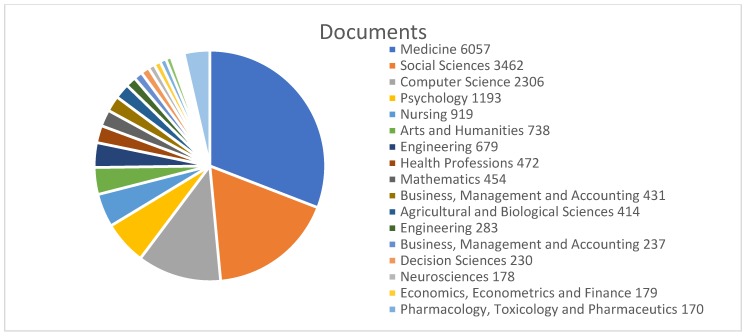
Distribution of scientific productions according to the main thematic areas.

The frequency of publications by each thematic area was acquired from the Scopus database. In Figure 8, the distribution of the main thematic areas is represented. This figure shows that the area with the highest percentage of documents was medicine (50.7%), followed by social sciences (28.9%), computer sciences (19.2%), and psychology (10%). Areas such as agricultural and biological sciences (1.3%); engineering (1.6%); or pharmacology, toxicology, and pharmaceutics (1.4%) were less common in the database. The “other” (5.9%) category represents unspecified areas.

These results show the two main thematic areas—medicine and social sciences (Table 4). Medicine is the central area of publication in the health field, as medicine is one of the most ancient areas of research [52]. The same could be said for social sciences, as social structures and social behavior have been studied for centuries [1].

The first quartile (Q1), Scimago Journal Rank (SJR), and Journal Citation Report (JCR) have been included in the table so as to present the importance and relevance of the major journals that have published more publications. These measures were chosen based on their quality and for being used worldwide in the scientific field. The quartiles are based on ranking each journal according to their subject, using the impact factor distribution the journal occupies for that subject category as a measure. In this sense, Q1 denotes the top 25% of the impact factor distribution. The Scimago Journal Rank measures the weighted citations received by the serial. Citation weighting depends on the subject field and the prestige of the citing serial. Finally, the Journal Citation Report is based on citations compiled from the Science Citation Index Expanded and the Social Sciences Citation Index [53].

The leading 11 journals that have published in this research field, and the number of publications in each according to the Scopus database, are shown in Table 5. As can be seen, most of the journals with the greatest number of documents published and the highest impact factors are from the United Kingdom (U.K.), Canada, and the United States (USA).

### 3.6. Determination of Scientific Groups and Utilization of Keywords

**Figure 9:**
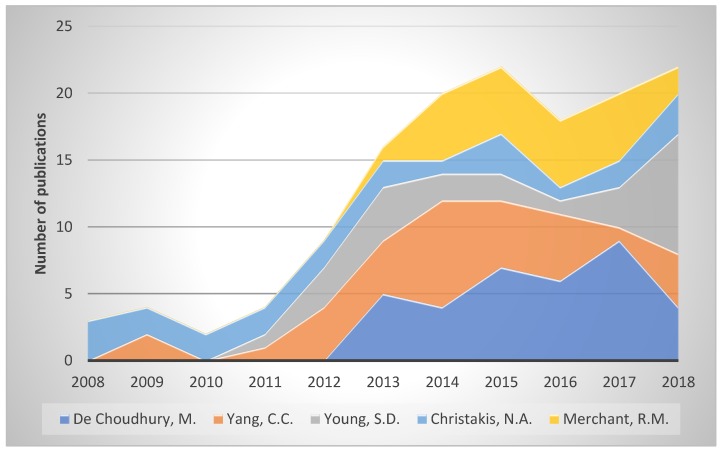
The top principal authors of the last decade.

A further analysis was carried out based on the dominant authors in the field of social networks related to the health of the young. Table 6 and Figure 9 represent the scientific productions of the top five researchers focused on this subject during the last decade. De Choudhury, M. tops this field, with 35 documents over 10 years. Nevertheless, this author has an h-index of 28, lower than Christakis, N.A., with an h-index of 71, and Merchant, R.M., with an h-index of 32. Following this, according to the h-index, was Yang, C.C. with 23 and Young, S.D. with 22. Although De Choudhury, M. has a lower h-index compared to Christakis, N.A. or Merchant, R.M., the total number of documents published by this author, 2776, is higher than for any of the other authors.

All of the authors with the highest frequencies of publication are from the United States. These results match the previous results, which showed the high impact and leading role of the United States in research on social media applied to the health field.

However, possible critical authors in the field of health and social media, such as Eysenbach, G., with an h-index of 44, were not included in the previous analysis, based solely on the number of documents they authored on this topic. Table 7 shows the 10 top authors of documents on this topic, with h-index, citations, total publications, and the year of the first publication included. Moreover, the author ID has been included so as to differentiate the authors with the same name, as any other researcher may access these details.

This table shows how younger authors have fewer publications, a lower h-index, and fewer publications. This is important to highlight, as the number of publications in this topic is not fully representative of the relevance of the authors.

Like any community, the scientific community is deeply connected, creating an interactive and dynamic network. This type of community usually has a central nucleus that is cohesively connected to other elements from the community that are less representative. The scientific community is generally replicated by clusters from other groups.

Clustering is a significant issue in the current work. Recognizing these groups has relative importance to the topic of study, as determining them makes it possible to define the quantity and quality of the existing associations between the authors of different institutions and areas of knowledge. The existence of interactions between different thematic areas, such as medicine and engineering, has been established [54]. The algorithmic mapping technique used by the software VOSviewer [41] was applied in order to identify and measure the association between authors. VOSviewer’s algorithm focused on the detection of items in a low-dimensional space, so that the distance between two items is a precise indicator of their affinity.

**Figure 10:**
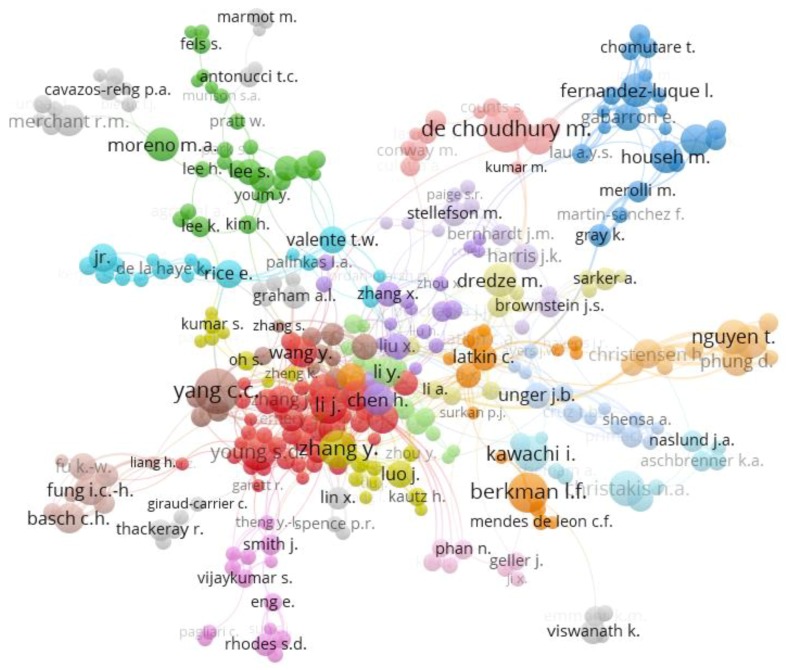
Scientific clusters of researchers focused on social networks in health.

Figure 10 depicts the clusters of the scientific communities of the authors. This figure displays the interactions between the principal authors and remaining researchers in the field of social networks related to the health of young people. The first cluster, led by Young S.D., is the greatest, with 43 authors. The following cluster (green) comprises 27 authors, of which the top author is Moreno, M., with 21 documents. The top author in collaborations and publications is De Choudhury, M., with 35 publications and 22 collaborators. On this basis, the second author is Yang, C.C., with 434 publications and 34 collaborators.

Another analysis we carried out was the determination of the keywords used in the publications in this field. During the last four decades, from the 11,966 documents found, the most common author keywords used were “human/s”, utilized in 10,936 items, followed by “social media” (3937 items), and “article” (3561 items). Table 8 illustrates the 40 most important keywords used in relevant documents during the last four decades.

The analysis of the authors’ keywords showed that most of the relevant keywords are commonly utilized for this topic. Nevertheless, it is essential to highlight that the term “human/s” was probably used to differentiate from animal research, rather than because of significance to the topic.

Based on these keywords, the results might imply the transversal inclusion of social media in the health field, from mental health to diabetes. However, it is essential to highlight that the keywords and topics of the studies also represented different points of view, such as on the side effects of using social media [55].

**Figure 11:**
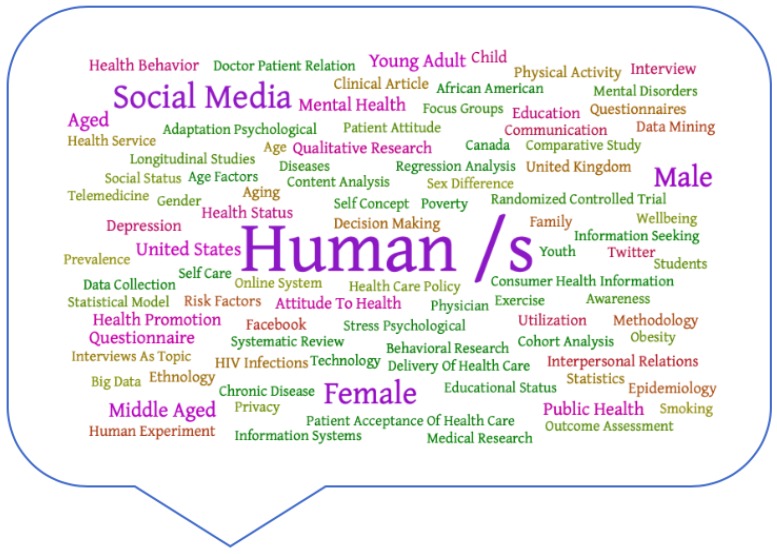
Cloud of the main keywords focused on social networks related to the health of young people.

Overall, the study of keywords in scientific works is highly relevant, as this determines the trends of publications and the follow-up of these publications. In this sense, Table 7 shows how similar concepts are often written differently; for example, “social media”, “Internet”, or “adolescent”. Figure 11 depicts a cloud of words, where the dimension of each word represents the significance of the keyword related to the number of documents in which it is used. The increased use of the term “social media” may be related to the increased use of platforms such as Facebook, Twitter, or Instagram [31,35,56]. The growth of other words, such as “health promotion” or “eHealth”, might be related to the development of telemedicine and studies focused on new technologies and health [57,58,59].

**Figure 12:**
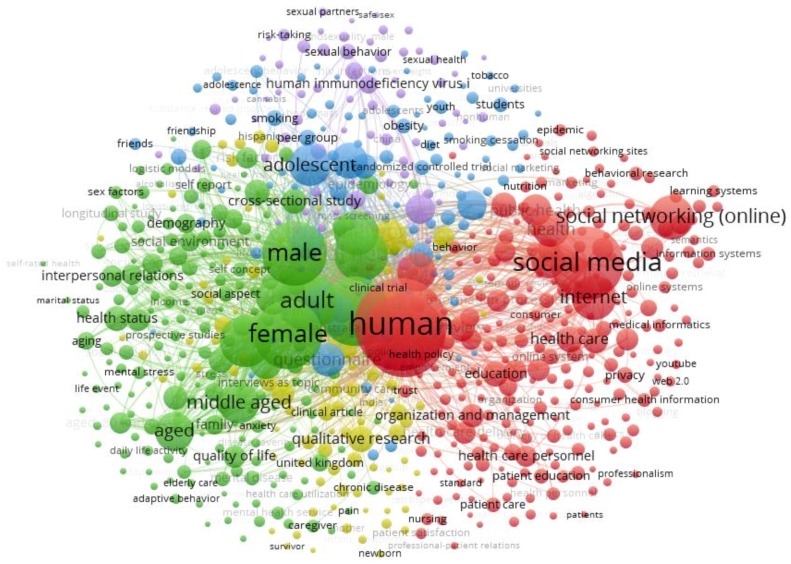
Clusters according to the co-occurrence of keywords.

Figure 12 displays the map of co-occurring keywords selected by researchers from the documents we analyzed that focused on social networks and the health of young people. The VOSviewer software with the Vos mapping technique was used to develop Figure 12. Each color symbolizes the separation between keywords, concerning the thematic area for which these colors have been selected. In addition, the dimension of the circles displays the frequency of use of each word, and the lines linking each circle show the associations among the different keywords used in the publications.

In this analysis, “human”, “social media”, and “article” are the most commonly used words. Table 8 shows the essential keywords used by the five top groups identified in the subject of social networks related to health and young people [60,61,62].

Social networks are used for multiple reasons related to health, including feedback, creating a support group, health interventions, or to determine the influence of these interventions [63,64,65]. Table 9 shows the main characteristics of the clusters from Figure 12, showing how the five clusters were found. The most important, the red cluster, focuses on social media and health education. The green cluster focuses on social networks and mental health, which matches the latest studies focused on preventing mental health problems in children and teenagers [66,67,68]. The third cluster focuses on how social media may play an essential role in prevention programs for adolescents. A comparison of the findings from this cluster with those of other studies confirms the important role that social media may have in preventing health problems in younger people [67,69]. This is consistent with our earlier observations and previous research, which showed that social networks may be preventative and help-seeking tools for young people with mental health problems, such as drug use, depression, or addiction [70]. The fourth cluster points out the relationship between ethnicity and health aptitude from a qualitative perspective. These results reflect those of Sunil and Xu (2019), who found that ethnicity and cultural background play an important role in health [71,72]. The purple cluster is focused on young people and the relationship of social networks to sexual health. This influence has been previously studied as being both positive and negative; either being used as a health intervention in patients with human immunodeficiency virus (HIV), or studied as a factor that contributes to the increase of HIV [73,74,75].

Based on these connections, it could be concluded that the first objective of this study was accomplished, as the first, fifth, and sixth clusters focused on the positive use of these technologies in the health field in general. As previously stated, previous studies have corroborated the main perspectives of the recent trend of publications on using social media as education or prevention tools [76]. The second objective has been partially completed, as the second, third, and fourth clusters focused not only on young people, but also adolescents. These results may be because of the interconnection between being a young person and being an adolescent; young adults are between 18 and 24 years old, and are often partially included in the definition of adolescence [69].

## 4. Conclusions

This paper examines trends in research focused on social media related to health and young people, including prevalence, topics, global distribution, and the networks of researchers involved.

Although the trajectory of relevant research remained relatively stable over the first thirty years profiled in our analysis, a significant increase can be seen between 2003 and 2018, correlating with the popularity of online social networks, especially among young people [21]. In addition, during that decade, the idea of using technology for following or supporting patients at a distance emerged and increased [77]. In this sense, social media has been utilized for support systems for patients, such as cancer patients, or to receive feedback from patients [78,79].

It is also essential to highlight the types of research seen in the review. For example, in terms of the research from Scopus, most publications were original articles in the form of quantitative cross-sectional studies or controlled trials. Another significant result was the topics of the studies, which were based on keywords, and showed a variety of multiple sub-areas of the health field relating to social networks, such as mental health, education, or chronic diseases.

The second significant finding was that collaboration between authors and countries seems to be led by the United States, acting as the standard connection between countries and authors. Based on this, significant countries in terms of health prevention measures and the health system, as well as the number of inhabitants, might be linked to the prevalence of studies on the role of social networks in health interventions and as a risk factor [60,80,81].

Regarding the areas of studies undertaken in the field of social networks in health, the area of medicine (50.7%) stands out as the most relevant. As previously stated, the supremacy of this area might be related to its evolution and relevance [59].

This work also determined communities by using the collaborations between countries found in the bibliometric study. Five clusters were identified, with the most significant focused on the actual usability of the social networks for educational purposes. Moreover, these results have shown how most countries are connected to the United States. These results seem consistent with previous results in the health field about the leading role of the United States [52]. The clusters are formed by those countries with traditional political, historical, and economic relationships. In general, therefore, it seems that the use of social networks in the health field, especially for young people, continues to grow as a tool, particularly for educational purposes, in certain places.

Nevertheless, like any research, this study has limitations. One source of weakness in this study that could have affected the measurement of the data is the choice of keywords used to interrogate the databases. This research focused on including different terms for social networks, more than including other terms for young people, such as “youth”. This was primarily to avoid the possible inclusion of publications not focused on any human population, such as those with the keyword “regenerative youth”. Additionally, some critical authors in this topic have not been included, or their presence is less representative. Moreover, the study of keywords and, therefore, the topic of the documents, might not represent the totality of the research carried out in the health field, as the keywords used were not MeSH terms. Finally, the boolean operators used, which were “OR” and “AND”, may have included some publications with the terms of the search, though the topic of study was different. However, based on the sample size, the number of the publications with different topic would produce an insignificant change in the result obtained in this study.

Overall, these findings have significant implications for the understanding of how the future of healthcare may lead to using social media in education and communication with patients. Additionally, this bibliometric analysis adds to the literature by elucidating the growing importance of social networks in health research, both as a topic of study and as a means of supporting scientific collaboration. This may help to inform future investments in public health research and surveillance using these different data sources, which may be particularly relevant for young people, who are a traditionally “hard to reach” group [82]. The bibliometric visualizations also provide an accessible means of communicating the key findings to researchers, policymakers, and those working in public health.

## Figures and Tables

**Table 1:** Main areas of research on social networks related to health during the last 10 years.

Year	Relation with Health	Analysis	Positive/Negative Effect	Topic	Reference
2019	Cancer patients	Cross-sectional study	Positive outcome	Social networks as a means to improve young patients’ health	[25]
2019	Kidney patients	Cross-sectional study	Positive outcome	Social media to support adolescent patients with disease	[26]
2019	Health and fitness	Interviews	Positive outcome	Social media as a pedagogical tool to understand or improve the wellbeing of young women	[27]
2019	Health education	Bibliometric	Positive outcome	Social networks as a pedagogical tool for education	[28]
2018	Suicide	Case report	Side effect	Social media as a negative factor in mental health	[29]
2018	Impulsive behavior and addiction	Cross-sectional study	Side effect	Addition to social media in young men	[30]
2018	Social distress	Interviews	Side effect	Stress in social media and the psychology health of young people	[31]
2018	Midwife study	Interviews	Positive outcome	Social media as an educational tool to enhance young people	[32]
2017	Emotional distress	Cross-sectional study	Side effect	Social media as a factor related to emotional distress	[33]
2017	Healthcare	Report	Positive outcome	Social media as an educational tool in health care	[34]
2017	Sexual healthcare	Experimental	Positive outcome	Social media as a mean to communicate sexual health	[35]

**Table 2:** International collaborations in research on social media related to health and young people.

Cluster	Color	Countries	Geographic Area	%
1	Red	Netherlands–Denmark–Finland–Norway–Belgium–Poland–Sweden–Russia Federation	Nordic countries–East Europe–Russia	37.6
2	Green	United Kingdom–Australia–Hong Kong–China	United Kingdom–Australia–Asia	25.1
3	Blue	United States–Canada–Switzerland–South Africa	United States–Canada–Africa	24.2
4	Yellow	Spain–France–Italy–Germany–Colombia	Europe–Latin America	13.4
5	Purple	Cuba–Peru–Uganda–Ethiopia	Latin America–Africa	2.9
6	Pink	Japan–Nepal–Thailand–Vietnam	Asia	2.8

**Table 3:** Publications and keywords utilized by the top ten international institutions.

Affiliation	Country	Publications	Main Keywords Used		
			**1**	**2**	**3**
University of Toronto	Canada	158	Human/s	Female	Social media
The University of Sydney	Australia	157	Human/s	Social media	Female
University of Michigan, Ann Arbor	United States	155	Human/s	Article	Female
The University of North Carolina at Chapel Hill	United States	152	Human/s	Article	Female
University of Washington, Seattle	United States	143	Human/s	Social media	Female
University of Melbourne	Australia	140	Human/s	Social media	Article
Harvard Medical School	United States	132	Human/s	Article	Male
University of California, Los Angeles	United States	131	Human/s	Article	Female
Johns Hopkins Bloomberg School of Public Health	United States	126	Human/s	Female	Adult
University of California, San Francisco	United States	123	Human/s	Social media	Male

**Table 4:** Main thematic areas concerning the total number of scientific productions found from the analysis.

Subject Area	Documents
Medicine	6057
Social Sciences	3462
Computer Science	2306
Psychology	1193
Nursing	919
Arts and Humanities	738
Engineering	679
Health Professions	472
Mathematics	454
Biochemistry, Genetics, and Molecular Biology	431
Business, Management, and Accounting	414
Agricultural and Biological Sciences	283
Environmental Science	237
Decision Sciences	230
Neurosciences	178
Economics, Econometrics, and Finance	179
Medicine	6057
Undefined	151
Other	705

**Table 5:** Quartile, Scimago Journal Rank (SJR), and Journal Citation Report (JCR) of major worldwide journals.

Source	Quartile Score	SJR (2018)	JCR (2018)	Total Docs (2018)	Total Doc (3 Years)	Total Ref.	Total Cites (3 Years)	Cites/Docs (2 Years)	Country
*Journal of Medical Internet Research*	Q1	1.74	4.90	1281	2018	5419	3335	2.10	Canada
*Lecture Notes In Computer Science Including Subseries Lecture Notes In Artificial Intelligence And Lecture Notes In Bioinformatics*	Q4	0.28	1.06	22,590	63,930	445,801	68,303	1.06	Germany
*Social Science and Medicine*	Q1	2.03	3.08	509	1599	44,305	18,063	3.71	United Kingdom
*Plos One*	Q1	1.18	2.76	217,985	62,994	223,689	74,005	3.11	United States
*ACM International Conference Proceeding Series*	-	0.17	0.59	-	6788	53,752	1313	0.56	Canada
*Studies In Health Technology And Informatics*	-	2.03	0.25	1553	1599	16,001	2809	3.71	Germany
*BMC Public Health*	Q2	1.38	2.56	1322	3650	58,519	3335	2.94	United Kingdom
*BMJ Open*	Q2	1.32	2.37	13,753	7215	26,298	38,028	2.65	United Kingdom
*Computers in Human Behavior*	Q1	1.71	4.30	462	2247	6258	13,804	6.14	United Kingdom
*Conference On Human Factors In Computing Systems Proceedings*	-	0.30	-	-	1924	10,072	5621	2.92	United States
*Journal of Health Communication*	Q2	1.0	1.77	110	417	1972	1108	2.37	United States
*American Journal of Public Health*	Q1	2.51	5.38	611	1786	20,651	5861	3.12	United States

**Table 6:** Progress of the top five authors’ works during the last decade.

	De Choudhury, M.	Yang, C.C.	Young, S.D.	Christakis, N.A.	Merchant, R.M.	Total Documents
2008	0	0	0	3	0	3
2009	0	2	0	2	0	4
2010	0	0	0	2	0	2
2011	0	1	1	2	0	4
2012	0	4	3	2	0	9
2013	5	4	4	2	1	16
2014	4	8	2	1	5	20
2015	7	5	2	3	5	22
2016	6	5	1	1	5	18
2017	9	1	3	2	5	20
2018	4	4	9	3	2	22
Total Documents	35	34	25	23	23	140

**Table 7:** Top 10 authors published in the topic, with h-index, citations, and total publications.

Author	Publications	H-index	Total Citations	Total Publications	First Publication	Author ID
De Choudhury, M.	35	28	2776	97	2007	18433530100
Yang, C.C.	34	23	1959	191	2000	7407740308
Berkman, L.F.	28	97	43,285	348	1976	7005551894
Young, S.D.	25	78	1241	78	2009	34876005800
Christakis, N. A.	25	71	26,657	236	1985	7005400323
Kawachi, I.	25	113	51,266	1005	1988	7103096477
Merchant, R. M.	23	32	6441	127	1998	14028632100
Dredze, M.A.	22	35	4669	137	2003	14041686400
Fernandez-Luque, L.	21	18	1251	85	2006	35224861700
House, M.	20	16	865	146	2005	8667908000

**Table 8:** Forty critical keywords used in publications.

Order	Term	Documents	%
1	Human/s	10,936	91.4
2	Social media	3937	32.9
3	Article	3561	29.8
4	Female	3450	28.8
5	Male	3086	25.8
6	Adult	2665	22.3
7	Social network	2167	18.1
8	Social Support	2083	17.4
9	Social networks	1538	12.9
10	Social networking (online)	1535	12.8
11	Internet	1513	12.6
12	Adolescent	1387	11.6
13	Ageing	1346	11.2
14	Psychology	1208	10.1
15	Priority journal	1201	10.0
16	Aged	1187	9.9
17	Health	1065	8.9
18	Young adult	1064	8.9
19	United States	1004	8.4
20	Major clinical study	965	8.1
21	Procedures	964	8.1
22	Controlled study	900	7.5
23	Questionnaire	898	7.5
24	Mental health	800	6.7
25	Public health	799	6.7
26	Health promotion	717	6.0
27	Statistics and numerical data	706	5.9
28	Attitude to health	595	5.0
29	Health care	582	4.9
30	Qualitative research	572	4.8
31	Review	559	4.7
32	Social networking	541	4.5
33	Medical information	540	4.5
34	Health status	536	4.5
35	Cross-sectional study	534	4.5
36	Child	533	4.5
37	Education	527	4.4
38	Health behavior	527	4.4
39	Cross-sectional studies	507	4.2
40	Surveys and questionnaires	505	4.2

**Table 9:** Keywords most utilized by the six top communities identified in the topic of social networks related to the health of young people.

Cluster	Color	Main Keywords	Topic	%
1	Red	Human–social media–medical information–eHealth–health education–public health	Social media–education	33.8
2	Green	Social network–age–epidemiology–gender–mental health–social support–psychological aspects	Mental health	28.9
3	Blue	Adolescent–young adults–health behavior–health promotion	Adolescents–health	14.0
4	Yellow	Qualitative research–interview–health attitude–ethnicity	Qualitative–health attitude	13.1
5	Purple	Health risk behavior–HIV infection–prevalence-risk assessment	Risk–prevention	10.3
